# Baseline MELD Score Predicts Hepatic Decompensation during Antiviral Therapy in Patients with Chronic Hepatitis C and Advanced Cirrhosis

**DOI:** 10.1371/journal.pone.0071262

**Published:** 2013-08-01

**Authors:** Georg Dultz, Martin Seelhof, Eva Herrmann, Martin-Walter Welker, Mireen Friedrich-Rust, Gerlinde Teuber, Bernd Kronenberger, Michael von Wagner, Johannes Vermehren, Christoph Sarrazin, Stefan Zeuzem, Wolf Peter Hofmann

**Affiliations:** 1 Medizinische Klinik 1, Klinikum der Goethe-Universität, Frankfurt am Main, Germany; 2 Institut für Biostatistik und mathematische Modellierung, Goethe-Universität, Frankfurt am Main, Germany; 3 Interdisziplinäres Facharztzentrum Sachsenhausen, Frankfurt am Main, Germany; 4 POLIKUM Gesundheitszentren Berlin, Berlin, Germany; The University of Hong Kong, Hong Kong

## Abstract

**Background and Aims:**

In patients with advanced liver cirrhosis due to chronic hepatitis C virus (HCV) infection antiviral therapy with peginterferon and ribavirin is feasible in selected cases only due to potentially life-threatening side effects. However, predictive factors associated with hepatic decompensation during antiviral therapy are poorly defined.

**Methods:**

In a retrospective cohort study, 68 patients with HCV-associated liver cirrhosis (mean MELD score 9.18±2.72) were treated with peginterferon and ribavirin. Clinical events indicating hepatic decompensation (onset of ascites, hepatic encephalopathy, upper gastrointestinal bleeding, hospitalization) as well as laboratory data were recorded at baseline and during a follow up period of 72 weeks after initiation of antiviral therapy. To monitor long term sequelae of end stage liver disease an extended follow up for HCC development, transplantation and death was applied (240weeks, ±SD 136weeks).

**Results:**

Eighteen patients (26.5%) achieved a sustained virologic response. During the observational period a hepatic decompensation was observed in 36.8%. Patients with hepatic decompensation had higher MELD scores (10.84 vs. 8.23, p<0.001) and higher mean bilirubin levels (26.74 vs. 14.63 µmol/l, p<0.001), as well as lower serum albumin levels (38.2 vs. 41.1 g/l, p = 0.015), mean platelets (102.64 vs. 138.95/nl, p = 0.014) and mean leukocytes (4.02 vs. 5.68/nl, p = 0.002) at baseline as compared to those without decompensation. In the multivariate analysis the MELD score remained independently associated with hepatic decompensation (OR 1.56, 1.18–2.07; p = 0.002). When the patients were grouped according to their baseline MELD scores, hepatic decompensation occurred in 22%, 59%, and 83% of patients with MELD scores of 6–9, 10–13, and >14, respectively. Baseline MELD score was significantly associated with the risk for transplantation/death (p<0.001).

**Conclusions:**

Our data suggest that the baseline MELD score predicts the risk of hepatic decompensation during antiviral therapy and thus contributes to decision making when antiviral therapy is discussed in HCV patients with advanced liver cirrhosis.

## Introduction

Chronic hepatitis C virus (HCV) infection is a major health burden with more than 170 million infected individuals worldwide. Progression to liver cirrhosis is observed in 2–35% of the patients after 20–25 years of chronic infection and once liver cirrhosis is established, the cumulative 5-year risk to develop hepatocellular carcinoma (HCC) is estimated to be 17% [1], [2].

For more than one decade, available antiviral treatment consisted of a dual therapy with pegylated interferon alfa-2a or -2b (peginterferon) in combination with the guanosine analog ribavirin leading to sustained virologic response (SVR) rates in approximately half of the patients [3], [4]. Licensing of the new HCV protease inhibitors boceprevir and telaprevir, as part of a triple therapy for untreated HCV genotype 1 patients and those who failed previous treatment, represents a milestone in HCV treatment. Untreated patients undergoing triple therapy achieve significantly higher SVR rates (66–75%) as compared to those receiving the dual therapy alone (37–44%) [5], [6], [7]. Patients with a previous virologic relapse, partial response, or non-response to peginterferon and ribavirin also benefit when retreated with boceprevir or telaprevir-containing triple therapies [8], [9].

It is well established, that the presence of advanced fibrosis or compensated liver cirrhosis negatively influence a patient’s individual chance for achieving an SVR [10]. In turn, patients with advanced disease may benefit most from antiviral therapy since it was demonstrated in several long-term follow up cohort studies that SVR can prevent hepatic decompensation, development of hepatocellular carcinoma, and is associated with reduced overall mortality [11], [12], [13], [14]. Albeit still unsatisfactory, subanalyses of the pivotal boceprevir and telaprevir trials have shown that SVR rates for patients with advanced fibrosis and liver cirrhosis receiving triple therapy are higher as compared to those receiving peginterferon and ribavirin alone (52–62% vs. 33–38%) [5], [6].

In patients with more severe disease, e.g. patients with advanced cirrhosis and those on the waiting list for liver transplantation, successful antiviral therapy in selected cases may halt the progression of liver disease, can prevent HCV re-infection of the transplanted liver and subsequently leads to a decrease of post-transplant morbidity and mortality [15], [16], [17], [18], [19], [20]. However, SVR rates in those patients have been shown to be poorer (approximately 25%) and peginterferon and ribavirin in those patients is associated with potentially life-threatening side effects and discontinuation rates ranged from 20–100%.

Recently, preliminary data from the French early access program for telaprevir and boceprevir (CUPIC study) reporting antiviral efficacies and the occurrence of adverse events in more than 400 cirrhotic patients receiving antiviral triple therapy were presented [21]. About 38–48% of the cirrhotic patients experienced serious adverse events during the first 16 weeks of antiviral triple therapy and 8 patients died.

Thus, decision making for antiviral therapy in patients with (advanced) liver cirrhosis remains a clinical challenge facing the dilemma of increased SVR rates on the one hand and therapeutic schedules that are associated with increased complications and fatal outcomes on the other hand [22]. Moreover, predictive factors in cirrhotic patients that are associated with serious adverse events and/or hepatic decompensation during antiviral therapy are poorly defined.

The aim of this retrospective cohort study was to define baseline characteristics that can help to predict the risk for hepatic decompensation in HCV patients with advanced liver cirrhosis during antiviral therapy with peginterferon and ribavirin. Clinical events indicating hepatic decompensation (ascites, hepatic encephalopathy, upper gastrointestinal bleeding, hospitalization) as well as laboratory data were recorded at baseline and during a follow up period of 72 weeks.

## Patients and Methods

In this retrospective cohort study we investigated 68 consecutive patients with advanced liver cirrhosis due to hepatitis C infection that were treated with pegylated interferon alfa-2a or 2b and ribavirin between the years 2002 and 2010 at the outpatient liver clinic of the J. W. Goethe University Hospital in Frankfurt, Germany. Diagnosis of chronic hepatitis C infection was based on a positive HCV antibody screening test at least six months before treatment and detection of HCV RNA in the blood by a quantitative assay. Liver cirrhosis was diagnosed when typical clinical features, endoscopical findings, laboratory results, imaging results or results of liver biopsy or transient elastography were present.

This cohort of patients with advanced cirrhosis consisted of individuals who had an episode of hepatic decompensation in the medical history, were classified beyond Child-Pugh class A at baseline of therapy, or had laboratory abnormalities (e.g. severe thrombocytopenia) that would generally exclude the patient from interferon-based antiviral therapy.

### Ethics Statement

The present study was approved by the Ethics Committee of Goethe University Hospital in Frankfurt, Germany, and written informed consent was obtained from all patients. The study was conducted in accordance with the guidelines of the Declaration of Helsinki and the principles of Good Clinical Practice.

### Therapy and Data Gathering

Hepatitis C therapy was administered according to national and international treatment guidelines [23], [24]. Patients were treated with either pegylated interferon alfa-2a (180 µg subcutaneously once a week) or -2b (1.0–1.5 µg/kg body weight subcutaneously once a week) in combination with weight-based dosage of ribavirin (800 mg–1200 mg daily). Planned treatment durations ranged from 16 weeks to 48 weeks according to HCV genotype and on-treatment virologic response. No patient was treated for longer than 48 weeks. Dose reductions were done according to package insert recommendations of peginterferon and ribavirin and on a case-by-case basis at the physicians’ discretion.

Approximately one-third (36.8%) of the patients had an episode of hepatic decompensation in the medical history or was classified as having liver cirrhosis beyond Child-Pugh class A by the initiation of therapy. The remaining patients had laboratory abnormalities indicating advanced cirrhosis (e.g. pronounced thrombocytopenia). Thus, the majority of patients were treated *off-label* as the label for antiviral therapy with peginterferons and ribavirin covers patients with well compensated Child-Pugh class A cirrhosis only. All patients gave informed consent prior to the initiation of therapy.

Clinical events indicating hepatic decompensation (ascites, hepatic encephalopathy, upper gastrointestinal bleeding, hospitalization) as well as laboratory data were recorded at baseline and throughout 72 weeks after initiation of antiviral therapy. These data were collected from electronic and paper files of the patients.

### Statistics

Data collection, data management, and statistical analyses were performed with SPSS software package, release 17.0 (SPSS Inc., Chicago, IL). To test for the association of patient parameters with the risk for hepatic decompensation, Mann–Whitney *U* tests were used. For the multivariate analysis backward stepwise logistic regression was used. The odds ratio and 95% confidence interval were calculated in order to determine the risk for hepatic decompensation under therapy. Findings were considered statistically significant at a p value <0.05 (two-sided).

## Results

Baseline characteristics of the 68 patients with advanced liver cirrhosis due to chronic HCV are summarized in Table 1. Forty-four (64.7%) of the patients were infected with genotype 1. The mean model of end stage liver disease (MELD) score at baseline was 9.19. Fourteen (20.6%) patients had experienced an episode of hepatic decompensation prior to the start of the antiviral treatment.

**Table 1:** Baseline characteristics of the study cohort.

	Patients (n = 68)
Age (years)	51.3 (28–69)
Sex (males), n (%)	37 (54.4)
HCV RNA (log_10_ IU/ml)	6.35 (2.34–7.21)
HCV genotype	
1, n (%)	44 (64.7)
2, n (%)	6 (8.8)
3, n (%)	13 (19.1)
4, n (%)	5 (7.4)
ALT (IU/L)	87 (17–349)
AST/ALT ratio	1.07 (0.45–2.67)
Platelet counts (/nl)	125 (33–281)
Leukocytes (/nl)	5.05 (1.47–13.67)
Hemoglobin (g/dl)	13.87 (7.8–17.6)
MELD score	9.19 (5–20)
Total bilirubin (mg/dl)	1.12 (0.3–4.4)
INR	1.19 (0.92–1.54)
Creatinine (mg/dl)	0.84 (0.46–1.93)
Previous decompensation, n (%)	14 (20.6)

Qualitative variables are shown in n (%) and quantitative variables are expressed as mean (range). AST, aspartate aminotransferase; ALT, alanine aminotransferase; MELD, Model for End-stage Liver Disease.

### Virologic Response and Discontinuation Rates

SVR was achieved in 18 (26.5%) of the patients (22.7% and 33.8% for genotype 1 and genotype non-1, respectively, p = 0.395, Table 2). The mean treatment duration was 38 weeks. In almost half of the patients (n = 33, 48.5%) treatment was discontinued early due to non-response (n = 23, 33.8%), breakthrough (n = 1, 1.5%) or side effects/hepatic decompensation (n = 9, 13.2%). In 6 of the 25 cases with hepatic decompensation the therapy was discontinued. In all other cases the patient’s condition could be stabilized with conservative treatment (e.g. diuretics, treatment for encephalopathy, antibiotics) so that the antiviral therapy could be continued. Completion of the planned therapy duration increased the chance for SVR significantly (full duration: 15 (50%) SVR, early discontinuation: 3 (7.9%) SVR, p<0.001). The minimum duration to achieve SVR was 16 weeks in one patient with genotype 2 infection. Although not statistically significant, a trend for an association between baseline MELD score and early therapy discontinuation was observed (p = 0.075).

**Table 2:** Treatment and decompensation parameters.

	Patients (n = 68)
Treatment duration (weeks)	37.9 (4–48)
SVR	18 (26.5)
Genotype 1	10 (22.7)
Genotype non-1	8 (33.3)
Decompensation	25 (36.8)
Ascites	18 (26.5)
Encephalopathy	4 (5.9)
GI-Bleeding	0 (0)
Hospitalization	19 (27.9)
Treatment discontinuation	33 (48.5)
due to Decompensation/side effects	6 (8.8)
Side effects	3 (4.4)
Nonresponse	23 (33.8)
Breakthrough	1 (1.5)

Qualitative variables are shown in n (%) and quantitative variables are expressed as mean (range). SVR, sustained virologic response.

The SVR rates of patients with or without hepatic decompensation did not differ significantly (28.0 vs. 25.6%, p = 0.829). The gamma-glutamyltransferase (GGT) levels at baseline were significantly lower in patients that achieved an SVR (73 vs. 114 U/l, p = 0.019). No association was documented between baseline MELD score and SVR (p = 0.227) or therapy duration (p = 0.104).

Most likely due to the relatively small patient number, no association between SVR and any other known baseline parameter including sex, age and viral load was observed.

### On Treatment Hepatic Decompensation

Hepatic decompensation during the 72-weeks period after initiation of antiviral treatment was observed in 25 cases (36.8%). In 18 patients (26.5%) development of ascites occurred, four patients (5.9%) experienced an episode of hepatic encephalopathy and 19 patients (27.9%) had to be hospitalized. Three of the hospitalized patients were admitted due to serious complications. One patient was hospitalized for treatment of severe pneumonia with sepsis. Two patients were hospitalized for acute portal vein thrombosis under antiviral treatment. In our patient collective no gastrointestinal bleeding was reported in the observational period. No patient died during the treatment or the follow-up period.

### Baseline Predictors of Hepatic Decompensation

In the univariate statistic analysis several baseline parameters differed significantly between patients with hepatic decompensation and patients without hepatic decompensation during the observational period (Table 3). Patients with hepatic decompensation had higher baseline MELD scores (11 vs. 8, p<0.001), mean bilirubin levels (1.56 vs. 0.86 mg/dl, p<0.001), INR (1.27 vs. 1.14, p = 0.001) and AST/ALT ratios (1.28 vs. 0.95, p = 0.005), as well as lower mean serum albumin levels (3.82 vs. 4.11 g/dl, p = 0.015), hemoglobin levels (13.28 vs. 14.25 g/dl, p = 0.044), platelets (103 vs. 139/nl, p = 0.014) and leukocytes (4.02 vs. 5.68/nl, p = 0.002) at baseline (Table 3). No association was observed for creatinine (0.87 vs. 0.82 mg/dl, p = 0.56), age (52.8 vs. 50.9 years, p = 0.80), treatment duration (37.5 vs. 38.1 months, p = 0.755) or gender (p = 0.99).

**Table 3:** Association of baseline parameters with hepatic decompensation in univariate and multivariate analyses.

baseline parameter	hepatic decompensation		Univariate p-value	Multivariate OR (95% CI) p-value
	yes	no		
**MELD score**	11	8	**<0.001**	**OR 1.56 (1.18–2.07), p = 0.002**
**Bilirubin [µmol/l]**	1.56	0.86	**<0.001**	n.s.
**INR**	1.27	1.14	**0.001**	n.s.
**Creatinine [mg/dl]**	0.87	0.82	0.563	–
**Platelets [/nl]**	103	139	**0.014**	n.s.
**Leukocytes [/nl]**	4.02	5.68	**0.002**	n.s.
**Albumin [g/l]**	38.2	41.1	**0.015**	n.s.
**Hemoglobin [g/dl]**	13.28	14.25	0.044	n.s.
**Age [years]**	51.84	50.93	0.798	–
**AST/ALT ratio**	1.28	0.95	**0.005**	n.s.

Baseline parameters are expressed as means. AST, aspartate aminotransferase; ALT, alanine aminotransferase; MELD, Model for End-stage Liver Disease; OR, odds ratio.

For the multivariate logistic regression analysis only those parameters were included that were significantly associated with hepatic decompensation in the univariate comparisons. In the multivariate analysis only the MELD score was shown to be independently associated with hepatic decompensation during antiviral therapy (p = 0.002) whereas all other parameters dropped out. The Odds ratio was calculated 1.56 (1.18–2.07), which shows that every increase in the MELD score of one point is associated with a 1.56-fold increased risk for decompensation (Table 3).

**Figure 1:**
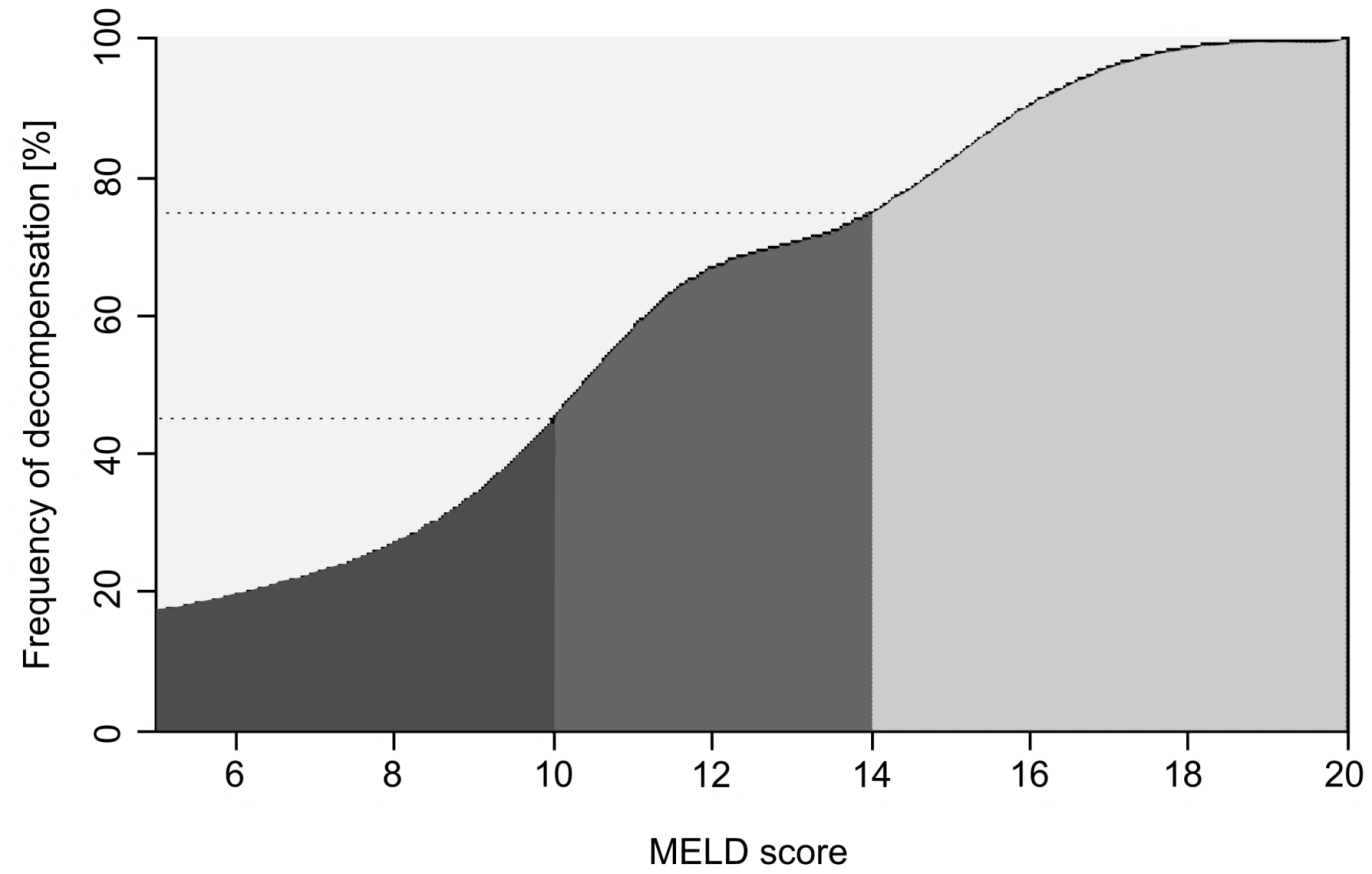
Frequency of decompensation according to the baseline MELD score. A low, moderate and high risk for decompensation in three MELD groups is shown (mean risk in each group was 22%, 59%, and 83%, respectively). The risk for decompensation during antiviral therapy increases with every increase in the baseline MELD score.

To better characterize the individual risk for hepatic decompensation, the patients were grouped according to their baseline MELD scores. Hepatic decompensation was observed in 22% (n = 10/54) of the patients with a MELD score of 6–9, in 59% (n = 10/17) of the patients with a MELD score between 10 and 13 and 83% (n = 5/6) of the patients with a MELD score above 14 (Figure 1).

### Extended Follow Up for HCC, Transplantation and Death

72 weeks of follow up after the start of antiviral treatment allow for the classification of treatment response and treatment related decompensation. To monitor long term sequelae of end stage liver disease an extended follow up for HCC development, transplantation and death was applied. Patients were followed for a mean period of 240 weeks (±SD 136 weeks) after initiation of antiviral therapy. HCC, transplantation or death occurred in 14 patients (20.6%). Five patients died (7.4%), five underwent liver transplantation (7.4%) and eight patients developed hepatocellular carcinoma (11.8%). None of the patients that achieved SVR needed liver transplantation or died in comparison to 20.0% of patients without SVR. Due to the small numbers the difference did not reach statistical significance (p = 0.052).

Baseline MELD score was significantly associated with the risk for transplantation/death (p<0.001) but not for the development of HCC (p = 0.231). Transplantation or death occurred in 2.2% of the low MELD patients (<10), in 35.3% of the medium MELD patients (11–13) and 50% in the high MELD patients (>13).

## Discussion

In HCV patients with bridging fibrosis or compensated liver cirrhosis SVR is clearly associated with an improved long-term clinical outcome. Hence, international treatment guidelines generally recommend antiviral therapy in this patient group [24], [25]. In individuals with more advanced disease, i.e. beyond Child-Pugh class A liver cirrhosis, individual decision making and treatment in an experienced liver clinic with a liver transplant program is recommended [26]. Successful antiviral treatment in patients with advanced cirrhosis may halt progression of liver disease or prevent HCV re-infection of the liver graft in those awaiting liver transplantation [22]. There are only few controlled studies available in patients with advanced or even decompensated liver cirrhosis. However, these studies provided evidence that SVR rates of less than 25% are achievable with peginterferon and ribavirin and that antiviral therapy is frequently associated with worsening of liver function and life-threatening side effects [15], [16].

In the present study, we retrospectively analyzed all patients with advanced cirrhosis who underwent antiviral therapy with peginterferon and ribavirin at our center between 2002 and 2010 (n = 68) and we assessed virologic efficacy, frequency of clinical events reflecting hepatic decompensation, and aimed to detect predictive parameters for patients at risk for decompensation. The overall SVR rate in our patient cohort was 26.5% and was comparable to other studies that assessed treatment outcomes in advanced cirrhosis patients [15]. The rate of hepatic decompensation defined as onset of ascites, hepatic encephalopathy, upper gastrointestinal bleeding or hospitalization was 36.8% in our cohort. The overall discontinuation rate was 48.5% including those who were discontinued due to hepatic decompensation and/or other serious adverse events (13.2%) and those who had a virologic non-response or breakthrough (35.3%). In previous studies, discontinuation rates ranged from 20–100% [22].

We retrospectively performed a multivariate analysis including a variety of baseline laboratory values as well as the MELD score to identify predictive factors associated with subsequent hepatic decompensation. Our data show that the baseline MELD score is independently associated with the risk for hepatic decompensation during antiviral therapy in our patient cohort. More detailed, every increase in the MELD score of one point increases the risk for hepatic decompensation 1.56 fold. When the patients were grouped according to their baseline MELD scores, hepatic decompensation occurred in 22%, 59%, and 83% of patients with baseline MELD scores of 6–9, MELD scores 10–13, and MELD scores >14. The MELD score was initially designed and established as a parameter to predict the 3-month mortality in patients who received a transjugular intrahepatic stent shunt and is currently used for the allocation of organ grafts in liver transplantation in the *United Network for Organ Sharing* (UNOS) and the *Eurotransplant* network [27]. In recent years, the MELD score has also been proven to predict perioperative mortality in patients with advanced liver disease [28], [29]. Our data suggest that the MELD score may also help to select patients with advanced liver cirrhosis in whom antiviral therapy with peginterferon and ribavirin is discussed.

The introduction of the HCV protease inhibitors boceprevir and telaprevir has substantially increased SVR rates in untreated HCV genotype 1 patients and those who failed previous treatment as compared to peginterferon and ribavirin alone [30]. Moreover, so-called difficult-to-treat patient groups including individuals with advanced fibrosis or liver cirrhosis achieve higher SVR rates with the triple therapy as compared to those receiving dual therapy. Thus, the motivation to treat patients with more advanced disease may rise in the near future. Data from the French early access program for telaprevir and boceprevir (CUPIC study) showed that 60–80% of the cirrhotic and prior non-responder patients were tested negative for HCV RNA early during treatment but serious adverse events occurred in 38–48% of all patients studied thus far and 8 patients died during the treatment period. This unexpectedly high frequency of serious adverse events differs from that seen in recent registration trials for boceprevir and telaprevir and raises important questions about safety and feasibility of antiviral treatment in cirrhotic patients. It is noteworthy, however, that approximately 30% of patients treated in the CUPIC study would not have been eligible for one of the boceprevir or telaprevir registration trials due to clinical or laboratory signs of advanced liver cirrhosis (beyond Child-Pugh class A) [21].

Our data show that already with a MELD score of 10 or higher the risk for decompensation under therapy is markedly increased. Nevertheless, this patient group should not per se be excluded from antiviral therapy with pegylated interferon and ribavirin. Under close clinical monitoring, deterioration of liver function and hepatic decompensation can be observed early and thus potential risks minimized. Treatment decision and supervision for this patient group should be in the hands of physicians with experience in the treatment of end stage liver disease and HCV therapy. Since the risk for decompensation in patients with a MELD score above 13 is even higher, treatment in this patient group should be considered in exceptional cases only. Treatment should ideally be reserved to transplant centers and even the listing for liver transplantation prior to the initiation of therapy should be considered.

Fortunately, we did not observe any therapy associated deaths in our patient cohort treated with dual therapy and hepatic decompensation could be managed by repeat clinical consultations and supportive treatment approaches in all patients. Earlier antiviral treatment studies have shown that infectious complications predominantly occur in advanced liver cirrhosis and antibiotic prophylaxis for patients with onset of ascites was proposed as a supportive treatment [31]. In the controlled trial by Iacobellis et al., the odds ratios were 2.95 for severe infection and 1.97 for dying from infection in treated decompensated patients with cirrhosis as compared to non-treated cirrhotic controls. Infections were associated with decreased liver function as expressed by the Child Pugh score and also by increasing MELD scores [15]. In line with this, a recent study by Roomer et al. showed that the neutrophile count of HCV patients during therapy cannot predict the risk for bacterial infections and dose reduction of peginterferon was not associated with a decrease of the infection rate [32].

The extended follow up period for the end points HCC, transplantation and death showed an association between MELD score and death/transplantation, as expected, demonstrating the integrity of our data. None of the patients with SVR needed liver transplantation or died during the follow up period which underlines the need for highly effective treatment regimes.

Currently, interferon-free treatment regimens evolve and may be available for many patients with chronic hepatitis C in the near future [33]. The combination of different classes of direct-acting antiviral agents, with or without ribavirin has been shown to result in high SVR rates in early clinical studies and showed dramatically better tolerability profiles as compared to interferon-based therapies [34], [35], [36], [37], [38]. Future studies are needed to show that interferon-free antiviral treatment is effective and feasible in HCV patients with advanced or decompensated liver cirrhosis.

In summary, our data showed that the baseline MELD score can be used to predict the risk for hepatic decompensation during peginterferon and ribavirin treatment in HCV patients with cirrhosis. Since there is accumulating evidence that severe complications in cirrhotic patients may occur more frequently during triple therapy with boceprevir and telaprevir, more studies are needed in larger cohorts to refine safety guidelines in these difficult-to-treat patients.
